# Changes in Brain Energy and Membrane Metabolism in Glioblastoma following Chemoradiation

**DOI:** 10.3390/curroncol28060424

**Published:** 2021-12-01

**Authors:** Astrid Ellen Grams, Stephanie Mangesius, Ruth Steiger, Ivan Radovic, Andreas Rietzler, Lisa Maria Walchhofer, Malik Galijašević, Julian Mangesius, Martha Nowosielski, Christian Franz Freyschlag, Johannes Kerschbaumer, Elke Ruth Gizewski

**Affiliations:** 1 Department of Neuroradiology, Medical University of Innsbruck, Anichstrasse 35, 6020 Innsbruck, Austria; astrid.grams@i-med.ac.at (A.E.G.); stephanie.mangeisus@i-med.ac.at (S.M.); ivan.radovic@icloud.com (I.R.); andreas.rietzler@i-med.ac.at (A.R.); malik.galijasevic@i-med.ac.at (M.G.); elke.gizewski@i-med.ac.at (E.R.G.); 2 Neuroimaging Core Facility, Medical University of Innsbruck, Anichstrasse 35, 6020 Innsbruck, Austria; 3 Department of Radiology, Medical University of Innsbruck, Anichstrasse 35, 6020 Innsbruck, Austria; lisa.walchhofer@kh-schwaz.at; 4 Department of Radiation Oncology, Medical University of Innsbruck, Anichstrasse 35, 6020 Innsbruck, Austria; julian.mangesius@i-med.ac.at; 5 Department of Neurology, Medical University of Innsbruck, Anichstrasse 35, 6020 Innsbruck, Austria; martha.nowosielski@i-med.ac.at; 6 Department of Neurosurgery, Medical University of Innsbruck, Anichstrasse 35, 6020 Innsbruck, Austria; christian.freyschlag@i-med.ac.at (C.F.F.); johannes.kerschbaumer@i-med.ac.at (J.K.)

**Keywords:** phosphorous magnetic resonance spectroscopy (31P-MRS), cerebral energy metabolism, ATP, glioblastoma, chemoradiation, normal-appearing brain tissue, tumor infiltration

## Abstract

Brain parenchyma infiltration with glioblastoma (GB) cannot be entirely visualized by conventional magnetic resonance imaging (MRI). The aim of this study was to investigate changes in the energy and membrane metabolism measured with phosphorous MR spectroscopy (31P-MRS) in the presumably “normal-appearing” brain following chemoradiation therapy (CRT) in GB patients in comparison to healthy controls. Twenty (seven female, thirteen male) GB patients underwent a 31P-MRS scan prior to surgery (baseline) and after three months of standard CRT (follow-up examination. The regions of interest “contrast-enhancing (CE) tumor” (if present), “adjacent to the (former) tumor”, “ipsilateral distant” hemisphere, and “contralateral” hemisphere were compared, differentiating between patients with stable (SD) and progressive disease (PD). Metabolite ratios PCr/ATP, Pi/ATP, PCr/Pi, PME/PDE, PME/PCr, and PDE/ATP were investigated. In PD, energy and membrane metabolism in CE tumor areas have a tendency to “normalize” under therapy. In different “normal-appearing” brain areas of GB patients, the energy and membrane metabolism either “normalized” or were “disturbed”, in comparison to baseline or controls. Differences were also detected between patients with SD and PD. 31P-MRS might contribute as an additional imaging biomarker for outcome measurement, which remains to be investigated in a larger cohort.

## 1. Introduction

Glioblastoma (GB) is a malignant tumor with infiltrating growth in the adjacent brain and is not always visible on conventional magnetic resonance imaging (MRI) sequences. Standard therapy consists of maximal possible gross total tumor resection followed by adjuvant concomitant chemoradiotherapy (CRT) [1].

During migration, glioma cells utilize myelin sheaths of adjacent neurons as a matrix for attachment [2]. In the early stages, invasive cells along the myelinated white matter pathways harm the pre-existing neural structures while leaving the white matter pathways undamaged [2,3,4]. Consequently, glioma cells can no longer be distinguished from pre-existing structures during infiltration [4], while increasing the cellularity, disturbing white matter fiber tracts, and altering cerebral metabolism. The presence of infiltrative malign cells in macroscopically inconspicuous areas distant from the tumor radiologically referred to as “normal-appearing white matter, was histologically proven in postmortem studies [5]. These alterations are detectable with diffusion tensor imaging (DTI) and magnetic resonance spectroscopy (MRS) [6,7,8]. 

With phosphorous-based MRS (31P-MRS), in vivo measurements of multiple functionally important phosphorus-containing metabolites can be performed, which, in neuroimaging studies, can be divided into two groups: energy-related metabolites (the energy metabolites inorganic phosphate (Pi), phosphocreatine (PCr), and adenosine triphosphate (ATP)), and cell membrane-related phospholipids (the mobile membrane phospholipid precursors phosphomonoesters (PME) and their breakdown products and intracellular signaling molecules phosphodiesters (PDE), which are related to membrane turnover). Furthermore, the PCr/ATP ratio is considered a surrogate for the energetic state of a tissue [9], the PCr/Pi ratio for the oxidative capacity [10,11,12], the Pi/ATP for ATP turnover [13,14], and PME/PDE ratios for membrane metabolism [15,16,17,18]. Ratios between the membrane-related and the energy-related metabolites (PME/PDE, PME/PCr, PDE/ATP, PDE/Pi, and PDE/PCr ratios) are regarded as a reflection of tumor growth [19]. 

Previous studies have described differences in energy and membrane metabolism detected with 31P-MRS between contrast-enhancing (CE) tumor and the contralateral hemisphere as well as brain tissue from healthy controls and further described changes in metabolites under therapy [19,20,21,22,23,24,25,26]. For example, Hattingen et al. suggested that metabolism of phospholipid cell membrane turnover is one of the major indicators for tumor growth, and that an elevated phosphoethanolamine (PEth) to glyceroethanolamine (GPE) ratio, as indicators for tumor malignancy and growth, could be a more sensitive marker of glioblastoma recurrence than is conventional MRI [20]. Walchhofer et al. demonstrated regional differences between energy metabolism in tumor areas and normal-appearing areas of the brain [27]. Galijašević et al. showed differences in 31-P MRS metabolites between GBs with distinct molecular characteristics [28]. Novak et al. used 31P-MRS to prove similarities in optic pathway gliomas and other low-grade gliomas, even though 1H-MRS and conventional MRI showed some high-grade characteristics, contrary to their later pathological diagnosis [29]. The aim of the present study was to investigate whether the energy and membrane metabolism in the CE tumor or normal-appearing brain tissue changes in the first months of CRT in GB patients, and whether this metabolism differs between patients with stable disease (SD) and progressive disease (PD). Accurate differentiation between SD and PD, as well as the development of bioimaging markers, for the purpose of differentiating between progression and pseudoprogression and between response and pseudo-response, is crucial in optimizing therapy and promptly reacting to potential changes in the pathophysiology of the tumor. Currently, a certain number of imaging modalities are available that help indicate these changes, but the invasive biopsy remains the gold standard.

## 2. Materials and Methods

### 2.1. Patients

**Figure 1:**
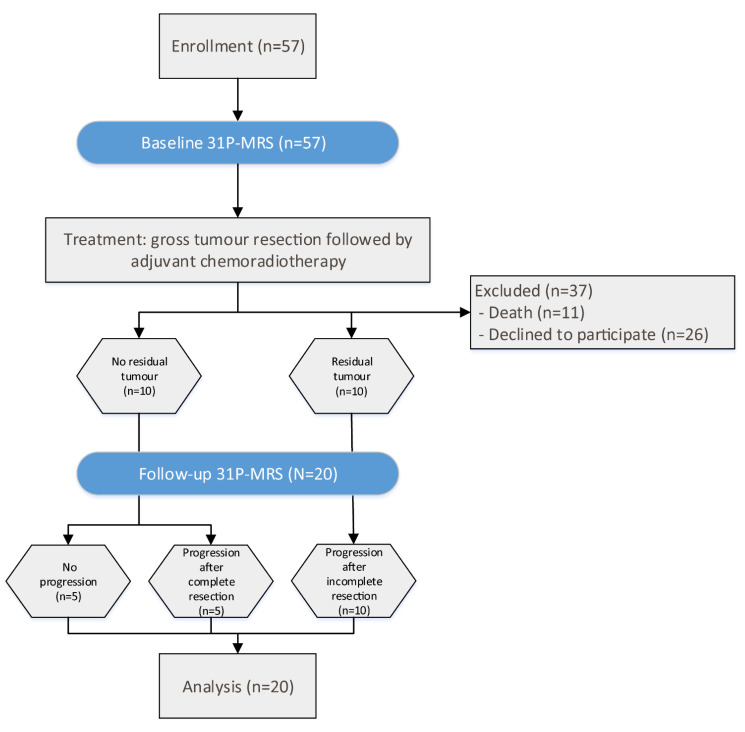
Flux diagram of patient selection and time flow of the study.

A total of 57 patients were prospectively enrolled in the study, after giving written informed consent. Of these patients, 37 did not receive a follow-up MRI scan (FU), either because of death (*n* = 11) or refusal to participate in the second scan (*n* = 26). Consequently, 20 patients (7 female and 13 male) with a mean age of 63 (range 36–77, median 64) years were included in the final analysis (Figure 1). This study was approved by the local ethics committee (AN 5100 325/4.19). Patients included in this study were newly diagnosed with GB, and underwent two MRI scans (including 31P-MRS) prior to surgery (baseline) and for the first staging scan (FU) after completion of chemoradiotherapy. The mean interval between the two scans was 4.05 (median 3.9) months. All patients underwent gross tumor resection and chemoradiotherapy, consisting of radiation therapy [30,31], with concomitant and adjuvant chemotherapy with temozolomide (TMZ), according to the Stupp regimen [1,32].

GBs were located in the temporal lobe (*n* = 5); parietal lobe (*n* = 5); frontal lobe (*n* = 3); occipital lobe (*n* = 2); and one each in the basal ganglia, fronto-temporal, fronto-parietal, temporo-parietal, and temporo-occipital. Of those, tumors in SD were located in the temporal (*n* = 2), temporo-occipital (*n* = 1), fronto-parietal (*n* = 1), and frontal (*n* = 1) lobes. Furthermore, tumors in PD were located in the parietal (*n* = 5), temporal (*n* = 3), occipital (*n* = 2), frontal (*n* = 2), temporo-parietal (*n* = 1), fronto-temporal lobe (*n* = 1), and basal ganglia (*n* = 1). Response assessment was performed by an experienced neuroradiologist, who reviewed all MRI scans according to the response assessment in neuro-oncology (RANO) criteria. PD is defined as a ≥25% increase in the cross-section area with a ≥40% increase in total volume, a new lesion, a significant or ≥100% increase in the volume of T2/FLAIR abnormalities, and SD as the best response for patients with non-measurable disease at baseline, with stable T2/FLAIR abnormalities [33]. 

In ten patients, no residual tumor was visible after surgery. In five of these patients, no tumor progression was observed in the FU scan. This group is referred to as “stable disease” (SD). In the other five patients with no residual tumor, the CE tumor was resected completely, but CE progression was visible in the FU scan, which is herein referred to as “progressive disease” (PD). In the remaining ten patients, residual CE tumor was detectable after surgery and was progressive in the FU scan of all five cases; it is also defined as PD (Figure 1).

31P-MRS data from age- and gender-matched controls in a cohort of 125 healthy volunteers were available (AN 5100 325/4.19 384/5.3 4213a).

### 2.2. 31P-MRS and Conventional MRI Scans

MRI scans were performed on a 3T MRI scanner (Verio, Siemens Medical AG, Erlangen, Germany). For the 31P-MRS sequence, a double-tuned 1H/31P volume head coil (Rapid Biomedical, Würzburg, Germany) was used. The 3D 31P-MRS block was planned on an isotropic T2-weighted 3D sequence, covering the entire cerebrum. Air-filled cavities, bone, fat, and boundary regions were spared, in order to avoid voxel contamination. The volume of interest was acquired with an 8 × 8 × 8 matrix, a field of view of 240 × 240 × 200 mm³, resulting in a 30 × 30 mm^2^ voxel size, and a slice thickness of 25 mm. The sequence was performed with a WALTZ 4 proton decoupling, a repetition time of 2000 ms, an echo time of 2.3 ms, a flip angle of 60°, and ten acquisitions for averaging. Similar variations of this sequence have already been performed in various studies [20,24,26,27,34,35,36,37,38]. 

Additionally, conventional MRI sequences were acquired with a 12-channel head coil (Siemens Medical AG, Erlangen, Germany) on the same scanner during the same session, including a transverse T1-weighted MPRAGE (voxel size: 0.9 × 0.7 × 1.2 mm³, TR: 1750 ms, TE: 3.3 ms, FA: 9°, FOV: 220 mm^3^, FOV phase: 71.9%, TA: 4:26 min) prior to and after injection of gadolinium-based contrast agent, and a transverse T2-weighted IR TSE (voxel size: 0.9 × 0.7 × 3.0 mm^3^, TR: 7060 ms, TE: 97.0 ms, FA: 150°, FOV: 220 × 187 mm^2^, base resolution matrix: 320 × 80%, number of slices: 49, TA: 5:12 min).

### 2.3. Data Post-Processing

**Figure 2:**
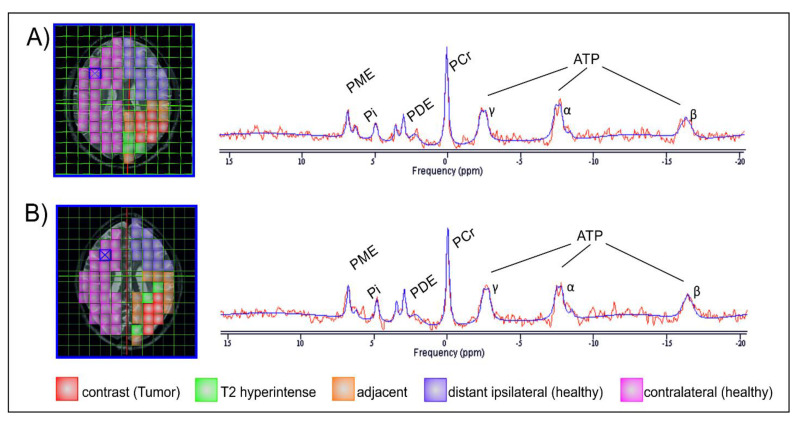
Images of one layer of the 31P-MRS measurement grid, each co-registered on an axial T2 space structural MR image of the same GBM patient, first at baseline (**A**) and subsequently 3 months after gross tumor resection and completion of chemoradiotherapy (**B**). Areas of interest are colored for better distinction. The two MRS spectra are exemplarily derived from the voxels highlighted with a blue X and depict the estimated individual metabolites of the MRS signal (red line) superimposed by the corresponding calculated fit (blue line).

31P-MRS .rda data files (Siemens Medical AG, Erlangen, Germany) were analyzed offline with jMRUI (version 5.0, MRUI Consortium, available at http://www.mrui.uab.es, accessed 1 October 2020). The fitting process performed with the non-linear square fitting algorithm AMARES [39] involved 12 Lorentzian-shaped exponentially decaying sinusoids representing metabolites (Figure 2, left to right), including phosphocholine and phosphoethanolamine (summarized as phsophomonoesters—PME), Pi, glycerophosphocholine and glycerophosphoethanolamine (summarized as phosphodiesters—PDE), PCr, and ATP. Two doublets (α-ATP and γ-ATP) and one triplet (β-ATP) were summarized, divided by three and designated ATP. 

The following regions of interest (ROI) were investigated separately at both MRI time points (Figure 2):

- contrast-enhancing (CE) tumor in the baseline scan and, if present, in the FU scan;
- areas adjacent to the CE tumor (AT) prior to therapy or the borders of the resection; area, including T2 hyperintense areas (which may represent either oedema or tumor infiltration);
- areas in the ipsilateral hemisphere, distant (ID) to the tumor or former tumor;
- contralateral (CL) brain.

The ROIs consisted of one or more 31P voxels, depending on the size of the ROI. If the GB was located in both hemispheres, the data from the contralateral brain were not included.

The number of voxels analyzed depended on the size of the area of interest and the spectral quality of the individual voxels. The voxels were assigned to the respective areas of interest by an experienced neuroradiologist (A.R.). Spectral quality was evaluated with an established method [40]. To minimize the effect of “voxel bleeding”, due to poor point spread function, only voxels with more than two-thirds of the tissue of interest were included.

The post-processing software provided unitless “areas under the curve” of the metabolites, from which the following ratios were generated: PCr/ATP, PCr/Pi, Pi/ATP, PME/PDE, PME/PCr, and PDE/ATP.

### 2.4. Statistical Evaluation

For statistical evaluation, data of individual voxels were assessed. Descriptive statistics were calculated with Excel (Microsoft 365, Microsoft, Redmond, WA, USA). Statistical analyses were performed with GraphPad Prism (Prism 8, GraphPad Software Inc., San Diego, CA, USA). Outliers were identified with the ROUT method and consequently excluded from the analysis. The normal distribution of metabolite ratios was assessed with the one-sample Kolmogorov–Smirnov test, applying a significance level of 5%. As the data were not normally distributed, the Kruskal–Wallis test was applied for group comparisons and multiple comparisons. SD and PD patients were compared using the Mann–Whitney U test. *p* values of ≤0.05 were defined as statistically significant.

## 3. Results

### 3.1. CE Tumour Areas

**Figure 3:**
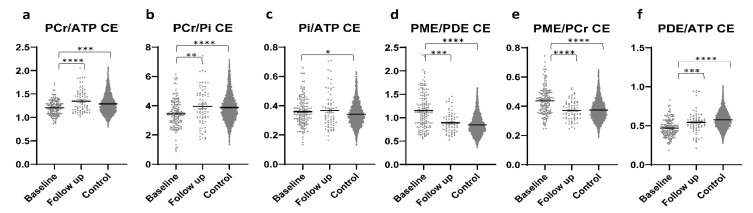
Different metabolite ratios (**a**–**f**) of energy and membrane metabolism (y-axis representing unit less metabolite ratios), measured with 31P-MRS, in the contrast-enhancing areas of patients with glioblastoma at baseline (154 voxels) and after approximately four months of standard therapy (70 voxels), as compared to the results for healthy controls (3030 voxels) (x-axis representing examined patient and control groups). Mean and standard error of mean (SEM) are depicted. Statistically significant differences in metabolite ratios Kruskal–Wallis test with post-hoc Dunn test are marked (* *p* < 0.05, ** *p* < 0.01, *** *p* < 0.001, **** *p* < 0.0001). Outliers were excluded from the analyses. Details on analyzed individuals, voxels, and outliers are given in Supplementary Table S1.

In patients with PD, significantly higher PCr/ATP and PCr/Pi ratios were found in CE tumor areas at the FU scan than in CE tumor areas at the baseline scan. While both ratios showed significantly lower values in comparison to controls at baseline, this difference was not present in the FU scans (Figure 3a,b). Significantly higher Pi/ATP ratios were found in CE tumor areas at baseline than in healthy brains, with no difference between FU scans and healthy brains or FU and the baseline scan (Figure 3c). The PME/PDE ratio was significantly higher at baseline than at FU or in healthy brains, with no difference between FU and controls (Figure 3d). The PME/PCr ratio was significantly lower in FU than at baseline and did not differ from controls (Figure 3e). The PDE/ATP ratio was significantly higher at FU than at baseline and significantly lower than in controls. In addition, this ratio was found to be significantly lower at baseline than in controls (Figure 3f). Outliers were excluded from the analyses.

### 3.2. Normal-Appearing Brain

#### 3.2.1. Areas Adjacent to the CE Tumor (AT)

**Figure 4:**
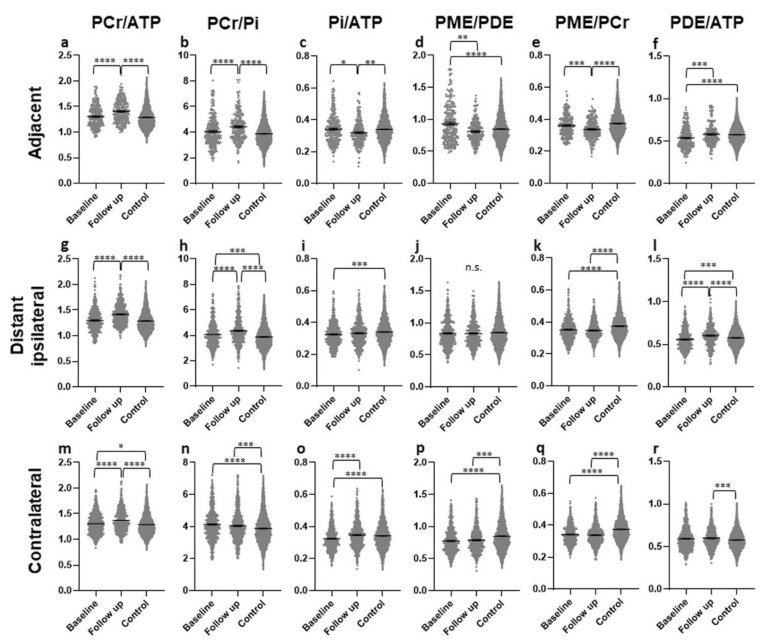
Different metabolite ratios of the energy and membrane metabolism (y-axis representing unitless metabolite ratios), measured with 31P-MRS, in different “normal-appearing” brain areas (adjacent (**a**–**f**), distant ipsilateral (**g**–**l**), contralateral (**m**–**r**)) of patients with glioblastoma at baseline (154 voxels) and after approximately four months of standard therapy (70 voxels), compared to the results for healthy controls (3030 voxels) (x-axis representing examined patient and control groups). Mean and standard error of mean (SEM) are depicted. Statistically significant differences in metabolite ratios assessed with the Kruskal–Wallis test and the post-hoc Dunn test are marked (* *p* < 0.05, ** *p* < 0.01, *** *p* < 0.001, **** *p* < 0.0001, n.s. not significant). Outliers were excluded from the analyses. Details on analyzed individuals, voxels, and outliers are given in Supplementary Table S2.

**Figure 5:**
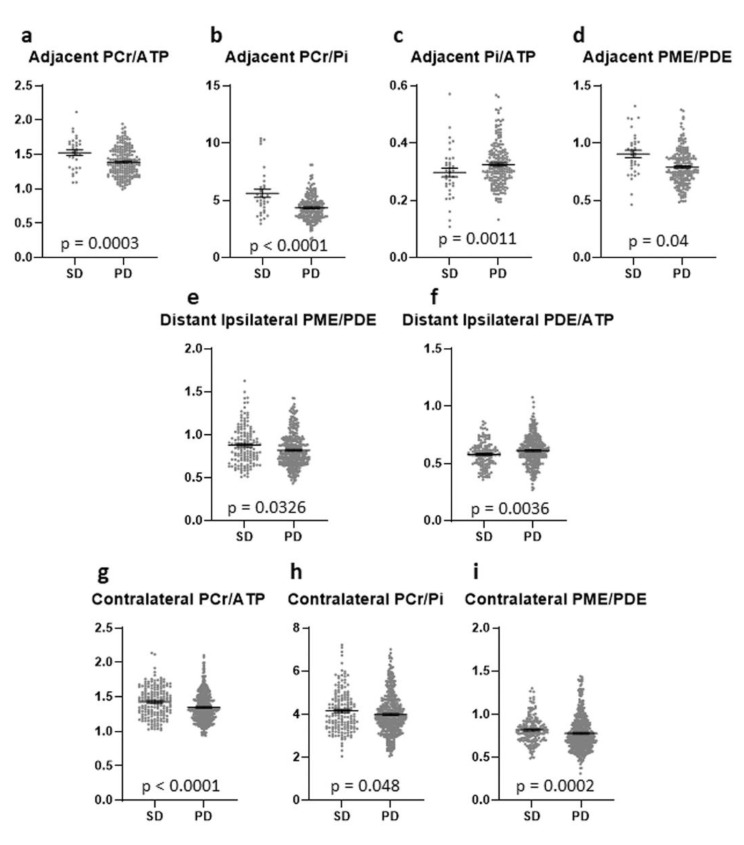
Different metabolite ratios of the energy and membrane metabolism (y-axis representing unit less metabolite ratios), measured with 31P-MRS, in different “normal-appearing” brain areas (adjacent (**a**–**d**), distant ipsilateral (**e**,**f**), contralateral (**g**–**i**)) of patients with SD (35 voxels) and patients with PD (200 voxels) (x-axis representing examined patient groups). The mean and standard error of mean (SEM) are depicted. Only statistically significant differences in metabolite ratios assessed with the Mann-Whitney U test are shown. Outliers were excluded from the analyses. Details on analysis individuals, voxels, and outliers are given in Supplementary Table S3.

In the AT ROIs, significantly higher PCr/ATP and PCr/Pi ratios were found in the FU scan than in baseline or controls, with no difference between baseline and controls (Figure 4a,b). Both ratios were higher in patients with SD than in patients with PD (*p* = 0.0003 & *p* < 0.0001) (Figure 5a,b). The Pi/ATP ratio was found to be significantly lower in the FU scan than in the baseline scan or in controls, with no difference between baseline and controls (Figure 4c). In patients with SD, this ratio was significantly lower than in patients with PD at FU (*p* = 0.0421) (Figure 5c). The PME/PDE ratio was significantly lower in FU than in baseline, with no differences between FU and controls (Figure 4d). The ratio was significantly higher in SD patients than in PD patients (*p* = 0.0011) (Figure 5d). The PME/PCr ratio was significantly lower and the PDE/ATP ratio was significantly higher in the FU scan than in the baseline scan or healthy brains (Figure 4e,f). The PDE/ATP ratio showed lower values at baseline than in controls, while the PME/PCr ratio revealed no differences between baseline and controls (Figure 4e,f). For both ratios, no difference was found between patients with SD and those with PD. Outliers were excluded from the analyses.

#### 3.2.2. Areas in the Ipsilateral Hemisphere, Distant (ID)

In the ID ROIs, the PCr/ATP and PCr/Pi ratios were significantly higher in the FU scan than in the baseline scan or controls (Figure 4g,h). The PCr/ATP ratio did not differ between baseline and controls, while the PCr/Pi ratio was significantly higher at baseline than in controls (Figure 4g,h). The Pi/ATP ratio was significantly lower at baseline than in controls, with no difference between FU and baseline or FU and controls (Figure 4i). None of these three ratios showed a difference between patients with SD and with PD. The PME/PDE ratio did not differ between any of the groups (Figure 4j), but was significantly lower in patients with PD than in patients with SD (Figure 5e). The PME/PCr ratio was found to be significantly lower at baseline and in the FU scan than in controls, with no difference between baseline and FU (Figure 4k). No difference was found between SD and PD. In addition, significantly higher PDE/ATP ratios in the FU scan were found than in the baseline scan or the controls, with significantly lower values in the baseline scan than in controls (Figure 4l). This ratio was significantly lower in patients with SD than in those with PD (*p* = 0.0036) (Figure 5f). Outliers were excluded from the analyses.

#### 3.2.3. Contralateral (CL) Brain

In the CL ROIs, the PCr/ATP ratios were significantly higher in the FU scan than in the baseline scan or in the controls, and were significantly higher in the baseline scan than in controls (Figure 4m). The ratio was significantly higher in patients with SD than in patients with PD (*p* < 0.0001) (Figure 5g). The PCr/Pi ratio was significantly higher at baseline and in the FU scan than in controls, with no difference between the baseline scan and FU (Figure 4n). This ratio was significantly lower in patients with PD than in those with SD (*p* = 0.048) (Figure 5h). Significantly higher Pi/ATP ratios were found in the FU scan than in the baseline scan, with no difference to controls (Figure 4o). In addition, significantly lower values were found at baseline than in controls (Figure 4o). For this ratio, no significant difference was found between patients with SD and those with PD. Significantly lower PME/PDE ratios were found in the FU and at baseline than in controls, with no difference between baseline and FU (Figure 4p). The ratio was significantly higher in patients with SD than in patients with PD (*p* = 0.0002) (Figure 5i). The PME/PCr ratio was significantly lower in the FU scan and at baseline than in controls, with no difference between baseline and FU (Figure 4q). The PDE/ATP ratio was significantly higher in the FU scan than in healthy controls, with no difference to the baseline scan or between baseline and controls (Figure 4r). For both ratios, no difference was found between patients with SD and with PD. Outliers were excluded from the analyses.

## 4. Discussion

The present study confirms differences in energy metabolism between CE tumor areas and healthy controls. 

Organisms and cells have evolved systems to modulate metabolic flux over short- and long-time scales [41]. Because metabolic needs can fluctuate on the order of seconds or persist for prolonged periods, regulatory circuits in a healthy system effectively control fluxes in a way that is adaptive for every situation [41]. These mechanisms of metabolic regulation seem to be altered in GB, resulting in increased metabolic processes, herein referred to as “disturbed”. It was seen that, in patients with residual or progressive tumor, the energy and membrane metabolism ratios in CE areas have a tendency to be “disturbed” under therapy, presenting higher values than for baseline or healthy brains. In the different “normal-appearing” brain areas of GB patients, the energy and membrane metabolism was either “normalized”, resembling values of healthy brains; “disturbed”; or did not differ to baseline. Some of these changes were more pronounced in patients with SD than in patients with PD. 

The PCr/ATP ratio has been described as a marker for the energetic state of a tissue [9], the PCr/Pi ratio for the oxidative capacity [10,11,12], the Pi/ATP ratio for ATP turnover [13,14], and the PME/PDE ratio as a surrogate for membrane turnover [15,16,17,18], and ratios between the membrane-related and the energy-related ratios have been described as a reflection of tumor growth [19]. 

To the best of our knowledge, there are no FU studies on 31P-MRS in patients with GB under standard therapy according to the Stupp regimen. One study investigated the antiangiogenic effect of bevacizumab on energy metabolism and found lower Pi/ATP ratios in CE tumor areas in responders than in non-responders, thus supporting the hypothesis that bevacizumab not only induces relative tumor hypoxia (T2’ decrease), but also affects energy homeostasis in recurrent GBM [26]. Furthermore, an increase in membrane metabolism in bevacizumab non-responders, in comparison to responders, was found in both CE tumor areas and normal-appearing brain tissue [20]. However, these studies are not directly comparable to the present one, as different ratios were investigated and patients included in our study received standard CRT according to the Stupp regimen without bevacizumab. Still, both studies are in accordance with the present study, in which standard therapy responders showed a decreased Pi/ATP ratio in the adjacent area as well. ATP catalysis results in the production of ADP and Pi. Therefore, decreased ATP/Pi in standard therapy responders could be a marker of increased ATP turnover, which is defined as the ratio of ATP content to ATP production [42]. The increase in this ratio could be due to either the increase in total ATP content or the decrease in ATP production. An in-vitro study by Lenz et al. showed increased sensitivity to the blockage of mitochondrial respiration in temozolomide-treated cells as compared to untreated cells [43]. GB is known as a tumor with mitochondrial dysfunction. This could lead to the preference for glycolysis over oxidative phosphorylation [44] and, consequently, to decreased ATP production. 

In the present study, lower PCr/ATP, PCr/Pi, and PDE/ATP ratios were found in CE areas than in brain tissue of healthy controls, which is in accordance with the literature [21,22,25,26]. In addition, we found an increase in PCr/ATP and PCr/Pi as well as a decrease in Pi/ATP in CE areas in the FU scan, as compared to the baseline scan. This may reflect a “disturbance” in the energetic state, the oxidative capacity, and the ATP turnover. As previously described [19,21,22,23,24,25,27,36], the Pi/ATP ratios were increased in the CE tumor areas at baseline as compared to those of controls, which persisted at FU, and may reflect a persistent increase in ATP turnover following therapy. PME/PDE and PME/PCr ratios showed decreasing values under therapy, resembling the values of controls, which may be an indicator of decreased membrane turnover and tumor growth.

In the present study, various changes in the energy and membrane metabolism were found in the “normal-appearing” brain tissue of patients with therapy-naïve GB on conventional MRI sequences, as compared to controls, which is in accordance with the existing literature [27]. In the AT areas, an increase in the energetic state (PCr/ATP) and oxidative capacity (PCr/Pi), a decrease in ATP turnover (Pi/ATP) and tumor growth (PME/PCr), as well as normalization of membrane turnover (PME/PDE), in comparison to baseline and healthy controls, occurred under therapy. This up- or down-regulation was more pronounced in patients with SD.

In the ID areas, an up-regulation of the energetic state (PCr/ATP) and the oxidative capacity (PCr/Pi) as well as a down-regulation of the tumor growth markers (PME/PCr and PDE/ATP) occurred under therapy and were more pronounced in patients with SD and less pronounced than in the AT areas. 

In the CL hemisphere, an up-regulation of the energetic state (PCr/ATP) was found, which was more pronounced in patients with SD. An increased oxidative capacity (PCr/Pi), as well as decreased membrane turnover (PME/PDE) and tumor growth markers (PDE/ATP & PME/PCr), persist between baseline and FU, as compared to controls. In patients with SD, PCr/Pi and PME/PDE ratios were higher than in patients with PD. In addition, a normalization of ATP turnover (Pi/ATP) was found in this area under therapy. 

Therefore, the present study underlines the results from earlier studies, suggesting that the cerebral energy and membrane metabolism in patients with GB is modified in the entire brain, in comparison to healthy brains. In addition, to the best of our knowledge, the present study is the first to find that cerebral energy metabolism is changed under short-term therapy; this effect is, to some extent, different between patients with SD and those with PD. However, a potential application of 31P-MRS as an outcome predictor needs to be investigated in a larger cohort.

This study has several other limitations inherent to the aggressive nature of the investigated tumor and the rapidly deteriorating health condition of our patients. 31P-MRS scans are time-intensive and, therefore, seriously intolerable for some GB patients. Consequently, 31P-MRS scans were only available for a small number of patients, especially at the time of FU, when we experienced a large number of dropouts. As GB still has a short survival time, even following state-of-the-art therapy, this limitation is difficult to overcome. The effect of “voxel bleeding” due to a poor point spread function is an omnipresent problem in MRS. We attempted to minimize this effect by choosing voxels in which the tissue to investigate was present in at least two-thirds of the voxel. However, the relatively low standard deviations and significant differences between the areas might prove the value of the presented results.

## 5. Conclusions

The present study underlines that the energy and membrane metabolism is modified in the entire brain of patients with GB and that it further changes under therapy. Observed changes also depend on the therapeutic success. 31P-MRS, together with other imaging and clinical parameters, might be an additional imaging biomarker for outcome measurement or therapy response, i.e., in the framework of radiomics studies. This could potentially pave the way for a more reliable and reproducible non-invasive diagnosis and more individualized therapy planning.

## Data Availability

All relevant data are contained in the article: The original contributions presented in the study are included in the article, further inquiries can be directed to the corresponding author. Anonymized data, not published in the article, can be shared on reasonable request from a qualified investigator after approval by the local ethics committee.

## Supplementary Materials

The following are available online at https://www.mdpi.com/article/10.3390/curroncol28060424/s1, Supplementary Table S1: Analysed individuals and analysed voxels (total voxels minus excluded outliers) reported in Figure 3, Supplementary Table S2: Analysed individuals and analysed voxels (total voxels minus excluded outliers) reported in Figure 4, Supplementary Table S3: Analysed individuals and analysed voxels (total voxels minus excluded outliers) reported in Figure 5.
