# Supplementary material for: Changes in Brain Energy and Membrane Metabolism in Glioblastoma following Chemoradiation
Source: Curr Oncol. 2021 Dec 1;28(6):5041–53. doi: 10.3390/curroncol28060424 (PMC8700426; doi:10.3390/curroncol28060424)
Supplement: Supplementary file 1 [file curroncol-28-00424-s001.zip › curroncol-1413552-supplementary.pdf]

Current Oncology

Supplementary Materials

Changes in Brain Energy and Membrane Metabolism in Glioblastoma Following Chemoradiation

Astrid Ellen Grams, Stephanie Mangesius, Ruth Steiger, Ivan Radovic, Andreas Rietzler, Lisa Maria Walchhofer, Malik Galijašević, Julian Mangesius, Martha Nowosielski, Christian Franz Freyschlag, Johannes Kerschbaumer and Elke Ruth Gizewski

Table S1. Analysed individuals and analysed voxels (total voxels minus excluded outliers) reported in Figure 3:.

		Individuals	Voxels	Outliers
**PCr/ATP**	**Baseline**	20	154	2
	**Follow up**	15	70	0
	**Control**	125	3030	8
**PCr/Pi**	**Baseline**	20	154	4
	**Follow up**	15	70	0
	**Control**	125	3030	33
**Pi/ATP**	**Baseline**	20	154	5
	**Follow up**	15	70	2
	**Control**	125	3030	27
**PME/PDE**	**Baseline**	20	154	1
	**Follow up**	15	70	3
	**Control**	125	3030	22
**PME/PCr**	**Baseline**	20	154	2
	**Follow up**	15	70	0
	**Control**	125	3030	7
**PDE/ATP**	**Baseline**	20	154	4
	**Follow up**	15	70	3
	**Control**	125	3030	13

Table S2. Analysed individuals and analysed voxels (total voxels minus excluded outliers) reported in Figure 4:.

		Individuals	Adjacent		Distant ipsilateral		Contralateral	
			Voxels	Outliers	Voxels	Outliers	Voxels	Outliers
**PCr/ATP**	**Baseline**	20	255	6	476	2	693	0
	**Follow up**	20	235	3	490	5	651	0
	**Control**	125	3030	8	3030	8	3030	8
**PCr/Pi**	**Baseline**	20	255	0	476	10	693	7
	**Follow up**	20	235	8	490	10	651	11
	**Control**	125	3030	33	3030	33	3030	33
**Pi/ATP**	**Baseline**	20	255	4	476	6	693	7
	**Follow up**	20	235	7	490	9	651	5
	**Control**	125	3030	27	3030	27	3030	27
**PME/PDE**	**Baseline**	20	255	0	476	4	693	2
	**Follow up**	20	235	5	490	9	651	1
	**Control**	125	3030	22	3030	22	3030	22
**PME/PCr**	**Baseline**	20	255	0	476	3	693	4
	**Follow up**	20	235	0	490	7	651	1
	**Control**	125	3030	7	3030	7	3030	7
**PDE/ATP**	**Baseline**	20	255	0	476	5	693	2

**Follow up**	20	235	9	490	4	651	0
**Control**	125	3030	13	3030	13	3030	13

Table S3. Analysed individuals and analysed voxels (total voxels minus excluded outliers) reported in Figure 5

		**Individuals**	**Adjacent**		**Distant ipsilateral**		**Contralateral**	
			**Voxels**	**Outliers**	**Voxels**	**Outliers**	**Voxels**	**Outliers**
**PCr/ATP**	**SD**	5	35	2	147	4	172	0
	**PD**	15	200	1	343	2	479	0
**PCr/Pi**	**SD**	5	35	2	147	3	172	5
	**PD**	15	200	2	343	9	479	9
**Pi/ATP**	**SD**	5	35	0	147	0	172	2
	**PD**	15	200	7	343	9	479	3
**PME/PDE**	**SD**	5	35	0	147	0	172	0
	**PD**	15	200	6	343	8	479	1
**PME/PCr**	**SD**	5	35	0	147	0	172	0
	**PD**	15	200	0	343	7	479	7
**PDE/ATP**	**SD**	5	35	0	147	0	172	0
	**PD**	15	200	3	343	4	479	4
